# Conjunctival Acute Graft-versus-Host Disease in Adult Patients Receiving Allogeneic Hematopoietic Stem Cell Transplantation: A Cohort Study

**DOI:** 10.1371/journal.pone.0167129

**Published:** 2016-11-30

**Authors:** Yao-Chung Liu, Jyh-Pyng Gau, Pei-Yu Lin, Catherine Jui-Ling Liu, Chia-Jen Liu, Jin-Hwang Liu, Nai-Wen Fan

**Affiliations:** 1 Division of Hematology, Department of Medicine, Taipei Veterans General Hospital, Taipei, Taiwan; 2 Faculty of Medicine, National Yang-Ming University, Taipei, Taiwan; 3 Department of Ophthalmology, Taipei Veterans General Hospital, Taipei, Taiwan; 4 Institute of Clinical Medicine, National Yang-Ming University, Taipei, Taiwan; University of Kentucky, UNITED STATES

## Abstract

**Background:**

To investigate the incidence, risk factors and survival of conjunctival acute graft-versus-host disease (aGVHD) in adult patients undergoing allogeneic hematopoietic stem cell transplantation (HSCT)

**Methods:**

This retrospective study included a total of 139 patients undergoing allogeneic HSCT between January 2012 and December 2014 at a tertiary referral hospital. Patients with ocular complaints after allogeneic HSCT or first donor lymphocyte infusion were evaluated by ophthalmologists. The risk factors for conjunctival aGVHD were analyzed using the Cox proportional hazards model. The overall survival was evaluated using Kaplan-Meier estimates.

**Results:**

Thirteen (9.4%) patients developed conjunctival aGVHD, including eight patients with pseudomembranous conjunctivitis. The cumulative incidence of conjunctival aGVHD was 2.1 cases per 10,000 person-day. The median age at HSCT was 47 years (range, 18 to 66) in all patients and 42 years (range, 24 to 58) in the 13 patients with conjunctival aGVHD. Median time of follow-up after allogeneic HSCT was 353 days (range, 11 to 1184). In univariate analysis, grades II-IV skin aGVHD (*P* = 0.002) and advanced systemic aGVHD except skin aGVHD (overall grades III-IV) (*P* = 0.001) were significant predictors for conjunctival aGVHD. In multivariate analysis, grades II-IV skin aGVHD was a significant risk factor (*P* = 0.04). The severity of conjunctival aGVHD was generally correlated with the systemic aGVHD (*P* = 0.001). Overall survival was significantly shorter in patients with grades II-IV aGVHD compared to those with grade 0-I (*P* = 0.01). Survival in patients with conjunctival aGVHD did not differ significantly from those without this complication (*P* = 0.94). In the subgroup analysis of patients with grades III-IV aGVHD, survival was significantly longer in patients with conjunctival involvement than those without (*P* = 0.03).

**Conclusions:**

The severity of conjunctival aGVHD is correlated with systemic aGVHD, but not with inferior overall survival.

## Introduction

Allogeneic hematopoietic stem cell transplantation (HSCT) can cure both benign and malignant hematological disorders, but is associated with many significant complications [1, 2]. Despite improvements in infectious prophylaxis, immunosuppressive treatment and supportive care, graft-versus-host disease (GVHD) remains a potentially lethal complication [3–6]. We recently observed that unexplained post-transplant pericardial effusion, a life-threatening complication, was a rare presentation of chronic GVHD (cGVHD) in adult HSCT patients [7]. We are also interested in exploring another rare post-transplant complication: conjunctival acute GVHD (aGVHD).

Ocular GVHD develops in 40–60% of patients receiving allogeneic HSCT, and significantly impairs their quality of life [8–12]. However, most ocular complications occur during the chronic stage. These include dry eye syndrome, corneal ulcers, cataract, glaucoma, cytomegalovirus (CMV) retinitis, fungal endophthalmitis, and acquisition of allergic conjunctivitis from atopic donors [8–11, 13–16].

There is limited research exclusively devoted to the prognosis of ocular aGVHD [17, 18]. Ocular findings in the acute stage include conjunctivitis, keratitis, dry eye, retinal hemorrhage, optic disc edema, anterior and posterior uveitis [19]. Of note, studies before 2000 reported that conjunctival involvement in aGVHD was an indicator for more severe systemic GVHD with high mortality [17, 18]. Given the altered clinical presentation of GVHD ascribed to profound advances in recent HSCT practice and post-transplant care, the assumption of conjunctival involvement as a poor prognostic factor needs to be re-evaluated. Accordingly, the main purpose of our clinical study was to elucidate the incidence, risk factors, and survival rate of conjunctival aGVHD patients after adult allogeneic HSCT.

## Materials and Methods

### Patients’ population

Adult patients receiving allogeneic HSCT between January 1, 2012 and December 31, 2014 in our institute were included. All patients were regularly followed up until May 1, 2015. Patients below age 18 were excluded. This study adhered to the tenets of the Declaration of Helsinki and was approved by the Institutional Review Board of the Taipei Veterans General Hospital, Taipei, Taiwan (VGH IRB no.:201411002CC). Informed written consent was waived by the approving IRB. In addition, patient records/information was also anonymized and de-identified prior to analysis.

After allogeneic HSCT, all patients underwent a comprehensive ocular evaluation by ophthalmologists for clinical ocular complaints with or without severe systemic aGVHD. Severity of aGVHD was graded according to the system of Glucksberg and Thomas. Severity of cGVHD was determined by NIH scoring system [20, 21]. Transplantation risk evaluation, detailed procedures of transplantation including conditioning regimens and GVHD prophylaxis and treatment were described in our previous report [7].

### Diagnosis and classification of conjunctival aGVHD

Systemic aGVHD and cGVHD were defined based on the National Institutes of Health (NIH) criteria [20]. As for the diagnosis of conjunctival aGVHD, patients met one of the following criteria:

1. Conjunctival complication within 100 days post allogeneic HSCT or donor lymphocyte infusion (DLI).
2. Conjunctival complication after 100 days post allogeneic HSCT or DLI in patients with systemic aGVHD
3. Conjunctival complication after 100 days post allogeneic HSCT or DLI in patients with overlap syndrome but acute manifestation is more severe than chronic.

The clinical staging of conjunctival aGVHD described by Jabs et al [17] was as follows: (1) stage 1, subtle conjunctival hyperemia without the evidence of an infectious etiology; (2) stage 2, conjunctival hyperemia associated with chemosis or serosanguinous exudate; (3) stage 3, pseudomembranous conjunctivitis; (4) stage 4, pseudomembranous conjunctivitis with corneal epithelial sloughing. Patients who presents with signs of adenoviral conjunctivitis, such as follicular conjunctivitis, mucopurulent discharge, watery eyes, were not diagnosed with conjunctival aGVHD.

### Treatment of conjunctival aGVHD

Topical steroids and topical prophylactic antibiotics were administered for patients presenting with conjunctival aGVHD. For stage 3 and 4 disease, pseudomembrane was frequently removed depending on the clinical condition; usually, every day or every other day for thick and rapidly growing pseudomembrane. When punctate keratitis or epithelial defects were noted, preservative-free artificial tears and ointment were prescribed. For patients with large corneal epithelial defect, therapeutic contact lenses were used to protect their corneas from irritation by inflamed conjunctiva and pseudomembrane.

### Statistical analysis

We used the nonparametric approach for the estimation of the cumulative incidence with accounting for competing risks. The risk factors for developing conjunctival aGVHD were analyzed using Cox proportional hazard models as compared with negative controls. Furthermore, Fine and Gray's proportional hazards model were used to evaluate the relationship between risk factors and the risk of conjunctival aGVHD and to estimate the subdistribution hazard ratios (HRs). For the analysis of risk factors, age 55 was adopted as a cutoff value based on the results of the receiver operating characteristic curve. The relationship between severity of aGVHD, conjunctival aGVHD and subsequent extensive cGVHD was analyzed using Jonckheere-Terpstra Test. The Kaplan—Meier product-limit method was used for evaluation of post-transplant survival of patients with systemic or conjunctival aGVHD. A Log-rank test was used to compare survival curves.

Factors with statistical significance (*P* < 0.1) upon univariate analysis were included in the multivariate analysis. All statistically significant levels were set at *P* < 0.05. Results were expressed as HR and their corresponding 95-percent confidence intervals (95% CI). All calculations were performed using the Statistical Package of Social Sciences software (version 18.0; SPSS, Inc., Chicago, IL, USA) and SAS statistical software (version 9.3; SAS Institute Inc., Cary, NC).

## Results

### Patient characteristics

Table 1 summarizes the characteristics of all patients. The median age at HSCT was 47 years (range: 18–66). Median time of follow-up duration after allogeneic HSCT was 353 days (range: 11–1184).

**Table 1:** Clinical characteristics of study patients (*n* = 139).

Patient characteristics	*n*	%	Mortality no.	Per 10,000 person-day
**Sex**				
Female	**66**	**47.5**	**27**	**8.9**
Male	**73**	**52.5**	**28**	**9.0**
**Age at allogeneic HSCT (year)**				
≤55	**105**	**75.5**	**36**	**7.3**
>55	**34**	**24.5**	**19**	**15.9**
**EBMT risk score**				
≤4	**105**	**75.5**	**34**	**6.8**
>4	**34**	**24.5**	**21**	**18.5**
**Indication for HSCT**				
AML/MDS	**70**	**50.3**	**30**	**11.2**
MPD	**2**	**1.5**	**0**	-
ALL	**19**	**13.7**	**3**	**2.8**
Lymphoma	**23**	**16.5**	**12**	**13.1**
MM	**12**	**8.6**	**8**	**15.2**
SAA	**9**	**6.5**	**1**	**1.6**
Others	**4**	**2.9**	**1**	**7.4**
**Transplant number(s)**				
One	**122**	**87.8**	**46**	**8.6**
Multiple	**17**	**12.2**	**9**	**10.9**
**Donor relation**				
Matched sibling	**54**	**38.8**	**23**	**8.2**
Alternative donors	**85**	**61.2**	**32**	**9.5**
**Conditioning regimen**				
A. Myeloablative	**84**	**60.4**	**33**	**8.8**
A1. TBI-based	**39**	**28.1**	**15**	
A2. Non-TBI-based	**45**	**32.3**	**18**	
B. Reduced-intensity	**55**	**39.6**	**22**	**9.1**
B1. TBI-based	**10**	**7.2**	**1**	
B2 Non-TBI-based	**45**	**32.4**	**21**	
**CMV infection prior conjunctival aGVHD**				
No	**45**	**32.4**	**14**	**7.0**
Yes	**94**	**67.6**	**41**	**9.8**
**Conjunctival aGVHD after allogeneic HSCT**				
Stage 1–2	**5**	**3.6**	**2**	**6.5**
Stage 3–4	**8**	**5.8**	**3**	**12.3**

aGVHD = acute graft-versus-host disease; ALL = acute lymphoblastic leukemia; AML = acute myeloid leukemia; CMV = cytomegalovirus; EBMT = European Group for Blood and Marrow Transplantation; HSCT = hematopoietic stem cell transplantation; MDS = myelodysplastic syndromes; MPD = myeloproliferative disorder; MM = multiple myeloma; SAA = severe aplastic anemia; TBI = total body irradiation.

**Fig 1:**
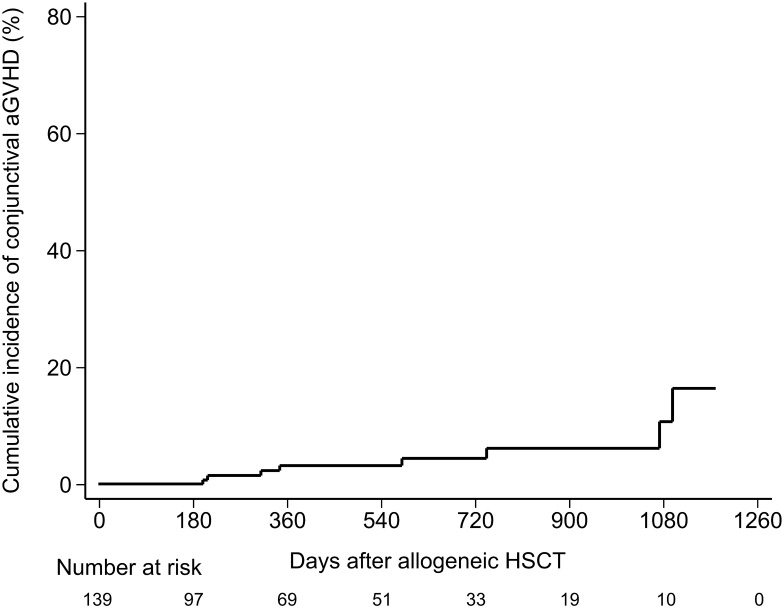
Cumulative incidence of conjunctival aGVHD after adjusting competing mortality for patients receiving HSCT. HSCT, hematopoietic stem cell transplantation.

During post-transplant follow-up, 60 patients developed aGVHD (43.2%). Of these, 13 (21.7%) patients experienced conjunctival aGVHD (Table 2). The cumulative incidence of conjunctival aGVHD was 2.1 cases per 10,000 person-day. Fig 1 showed the cumulative incidence after adjusting competing mortality. The median onset time was 64 days (range: 20–142) after HSCT, which was 15 days from the onset of the first presentation of systemic aGVHD (range: 3–74). Among the 13 patients, 8 patients developed pseudomembranous conjunctivitis. The median onset time of pseudomembrane formation was 68 days after transplants (range: 20–142), which was 15 days after the median onset of first manifestation of systemic aGVHD (range: 3–67).

**Table 2:** Characteristics of patients with conjunctival aGVHD after allogenic HSCT or DLI.

Patient	Age (years)/sex	Diagnosis	Systemic aGVHD (grade)	Conjunctival aGVHD (stage)	Conjunctival aGVHD detected after HSCT or DLI (days)	Follow-up after HSCT (days)	Outcome
1	57/F	Nasal NK T-cell lymphoma	IV	4	72	288	Died
2	35/F	AML	III	3	85	198	Alive
3	27/F	ALL	IV	3	33	207	Alive
4	24/F	AML	III	3	20	309	Alive
5	42/M	AML	III	3	142*	345	Alive
6	40/F	DLBCL	III	3	64	261	Died
7	59/F	AML	I	3	59	90	Died
8	58/M	AML	I	3	128*	741	Alive
9	57/F	AML	IV	2	109*	579	Alive
10	27/M	AML	III	1	112*	249	Died
11	24/M	ALL	III	1	52	1072	Alive
12	47/F	MM	III	1	41	1097	Alive
13	58/F	AML	II	1	56	67	Died

aGVHD, acute graft-versus-host disease; ALL, acute lymphoblastic leukemia; AML, acute myeloid leukemia; DLI, donor lymphocyte infusion; DLBCL, diffuse large B-cell lymphoma; HSCT, hematopoietic stem cell transplantation; MM, multiple myeloma; NK, natural killer

*Presence of chronic GVHD when conjunctival aGVHD was diagnosed: patient 5:no; patient 8–10: yes, but limited to skin/mucosa

### Outcome of conjunctival aGVHD

Of the 13 patients with conjunctival aGVHD, 11 patients (84.6%) developed overall grade II-IV systemic aGVHD. In the subgroup with pseudomembrane formation, 6 out of 8 patients (75%) had grade III-IV systemic aGVHD. In addition, using the analysis for each overall grade of aGVHD, there was a significant association between conjunctival aGVHD and the overall grade of aGVHD (Table 3, Jonckheere-Terpstra Test, *P* = 0.001). There was also a significant association between overall grade aGVHD and subsequent extensive cGVHD (*P* < 0.001).

**Table 3:** Relationship of severity of aGVHD, conjunctival aGVHD and subsequent extensive cGVHD.

	No. of patients	Conjunctival aGVHD; *n* (%)	Extensive cGVHD; *n* (%)
**Overall grade of aGVHD**			
**0**	79	0 (0)	4 (5.06)
**I**	22	2 (9.1)	7 (31.8)
**II**	4	1 (25)	0 (0)
**III**	25	7 (28)	7 (28)
**IV**	9	3 (33)	3 (33)
*P* value*		*P* = 0.001	*P*< 0.001

aGVHD = acute graft-versus-host disease; cGVHD = chronic graft-versus-host disease; No. = number (s)

*: Jonckheere-Terpstra Test

**Fig 2:**
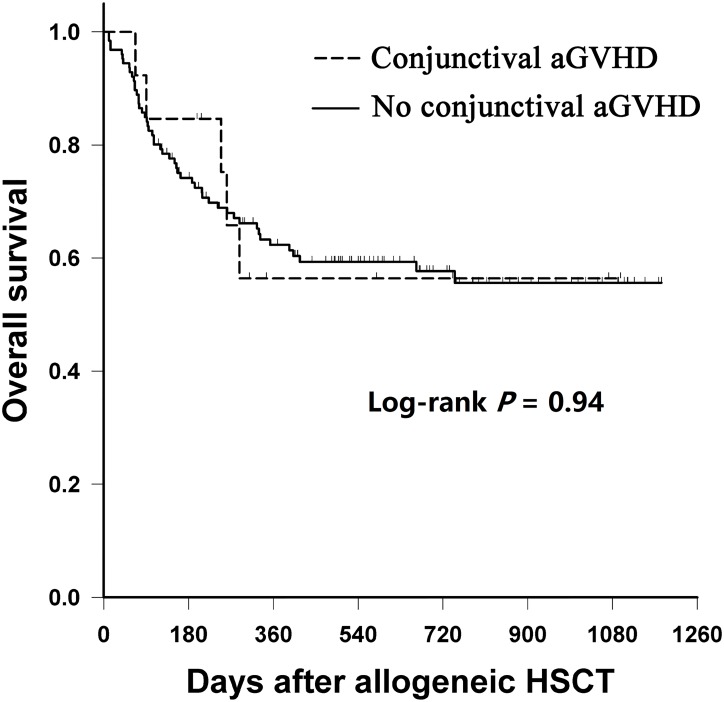
Overall survival after HSCT for patients developing conjunctival aGVHD or not. HSCT, hematopoietic stem cell transplantation; aGVHD, acute graft-versus-host disease.

**Fig 3:**
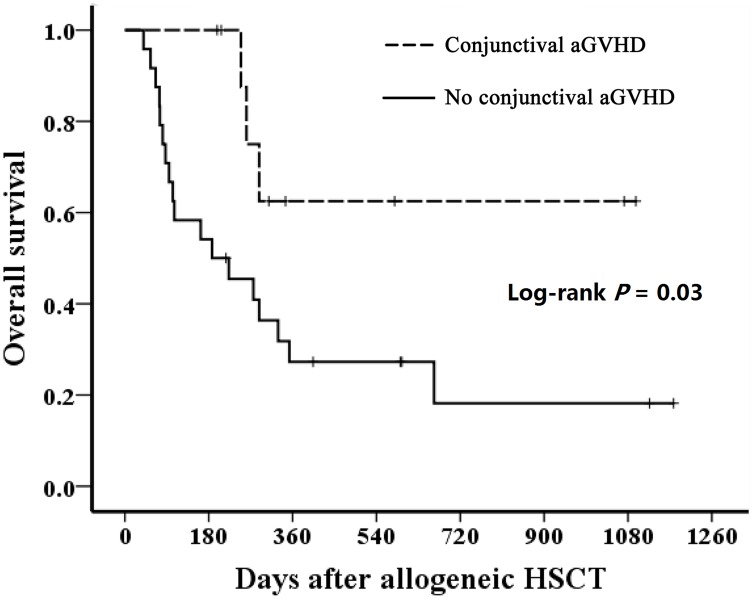
Overall survival after HSCT for systemic grade III-IV aGVHD patients developing conjunctival complication or not. HSCT, hematopoietic stem cell transplantation; aGVHD, acute graft-versus-host disease.

Eight patients (61.5%) with conjunctival aGVHD survived to date. Four patients died of subsequent infection and the other one died of aGVHD with hepatic failure. The post-transplant median survival of patients with conjunctival aGVHD was 288 days and 275 days in the subgroup with pseudomembrane formation. Using Kaplan-Meier analysis, overall survival was significantly shorter in patients with grades II-IV aGVHD compared to those with grades 0-I (log-rank *P* = 0.01). However, survival in patients with conjunctival aGVHD did not differ significantly from those without the complication (log-rank *P* = 0.94; Fig 2). In the subgroup analysis of patients with grades III-IV aGVHD, survival was significantly longer in patients with conjunctival involvement compared with those without. (log-rank *P* = 0.03; Fig 3).

### Risk factors for conjunctival aGVHD

Thirty-four of the 139 patients were more than the age of 55 years with high European Group for Blood and Marrow Transplantation (EBMT) risk scores (risk scores > 4) [22], of whom 5 patients (4.4 cases per 10,000 person-day) developed conjunctival aGVHD. In univariate analysis, age > 55 years (HR: 2.570; 95% CI: 0.839–7.873; *P* = 0.09) and EBMT risk scores > 4 (HR: 2.721; 95% CI: 0.850–8.712; *P* = 0.09) showed a trend of significance. In multivariate analysis, there was no significant difference between these two subgroups. The analysis is detailed in Table 4. By analyzing the impact of factors including sex, disease type upon diagnosis, the number of transplants, donor type, conditioning regimen and post-transplant CMV infection prior to conjunctival aGVHD, no statistical significant difference was found. High grade skin aGVHD (HR: 5.983; 95% CI: 1.928–18.567; *P* = 0.002) and advanced systemic aGVHD (grade III-IV) except skin involvement (HR: 9.435; 95% CI: 2.582–34.476; *P* = 0.001) were significant predictors for conjunctival aGVHD in the univariate analysis. However, in multivariate analysis, aGVHD with skin involvement ≥ grade II was an independent significant risk factor for the occurrence of conjunctival aGVHD (HR: 5.254; 95% CI: 1.044–26.454; *P* = 0.04). Fine and Gray subdistribution hazard regression models, with death as competing event, showed that grades II-IV skin aGVHD retained a trend of significance in multivariate analysis. (HR: 6.449; 95% CI: 6.449–57.944; *P* = 0.096; S1 Table)

**Table 4:** Risk factors for conjunctival aGVHD after adult allogeneic HSCT.

Factors	*No*. *of patients*	*Conjunctival aGVHD*		Univariate analysis			Multivariate analysis		
		*n*	Per 10,000 person-day	*HR*	*95% CI*	*P* value	*HR*	*95% CI*	*P* value
**Age at HSCT**									
≤ 55	105	8	1.6						
> 55	34	5	4.2	2.570	0.839–7.873	0.098	2.444	0.665–8.985	0.179
**EBMT risk score**									
≤ 4	105	8	1.6						
> 4	34	5	4.4	2.721	0.850–8.712	0.092	1.356	0.353–5.217	0.657
**Sex**									
Female	66	9	3.0						
Male	73	4	1.3	0.448	0.137–1.458	0.182			
**Disease type**									
Myeloid	78	8	2.6						
Non-myeloid	61	5	1.6	0.542	0.170–1.724	0.300			
**Conditioning**									
Reduced-intensity	55	5	2.1						
Myeloablative	84	8	2.1	1.100	0.354–3.417	0.869			
**Conditioning**									
Other	90	4	1.5						
TBI-based	49	9	2.5	1.605	0.493–5.225	0.432			
**Conditioning**									
Other	101	9	1.9						
Fludarabine-based	38	4	2.7	1.245	0.376–4.117	0.720			
**Transplant no.**									
One	122	12	2.2						
Multiple	17	1	1.2	0.427	0.053–3.428	0.424			
**Donor type**									
Matched sibling	54	5	1.8						
Non-matched sibling	85	8	2.4	1.954	0.597–6.394	0.268			
aGVHD grade*									
0-II	106	5	1.0						
III-IV	33	8	6.7	5.983	1.928–18.567	0.002	2.254	0.507–10.014	0.285
**aGVHD of skin**									
Stage 0-I	101	3	0.6						
Stage II-IV	38	10	6.6	9.435	2.582–34.476	0.001	5.254	1.044–26.454	0.044
**CMV infection**									
No	45	3	1.5						
Yes	94	10	2.4	1.685	0.462–6.149	0.430			

aGVHD = acute graft-versus-host disease; CI = confidence interval; cGVHD = chronic GVHD; CMV = cytomegalovirus; EBMT = European Group for Blood and Marrow Transplantation; GVHD = graft-versus-host disease; HR = hazard ratio; HSCT = hematopoietic stem cell transplantation; No. = number (s); TBI = total body irradiation. Factors with statistical significance (*p* < 0.1) upon univariate analysis were included in multivariate analysis

*:overall grade of aGVHD except skin involvement

## Discussion

The cumulative incidence of conjunctival aGVHD was 2.1 cases per 10,000 person-day. The proportion (13/139) of conjunctival aGVHD in our cohort is consistent with the results of previous reports (7.2%-17%) [16–18]. All of our patients developed conjunctival complications during episodes of systemic aGVHD, which was also in line with previous findings [18, 23]. In our cohort, the 15 days median onset time of conjunctivitis after the first manifestation of systemic involvement was similar to a previous report (14 days) [17].

The organs frequently involved in aGVHD include the skin, gastrointestinal tract and liver. The skin is most frequently affected and is usually the earliest organ involved [6]. The occurrence of ocular aGVHD following skin aGVHD is probably just a reflection of the different nature of skin and conjunctiva in their susceptibility to the development of aGVHD. In the literature, the severity of conjunctival aGVHD generally correlates with the severity of systemic disease and higher mortality rates [17–18, 24–25]. Hirst et al. reported that 5 (83.3%) of 6 patients with pseudomembranous conjunctivitis in the prospective subgroup died during the acute stage [18]. Jabs et al. reported that 17 (89.5%) of 19 patients with conjunctival aGVHD died at a median time of 76 days after transplantation [17]. In our cohort, there was a tight correlation in severity between conjunctival aGVHD and systemic aGVHD. However, overall survival was not influenced by the occurrence of either conjunctival aGVHD or pseudomembranous conjunctivitis. In patients with advanced systemic aGVHD, those with conjunctival involvement had superior survival than those without. In another scenario, conjunctival aGVHD associated with advanced systemic aGVHD may be manageable. The marked ocular involvement may prompt physicians to give more active treatment to control the aGVHD, especially in patients with severe aGVHD, which then may prolong some patients’ survival.

The present study found that ≥ grade II skin aGVHD is a significant risk factor for developing conjunctival aGVHD. Likewise, one study showed that prior acute skin GVHD was associated with a higher incidence of ocular GVHD in univariate analysis, and retained a trend of significance in multivariate analysis [26]. Westeneng et al reported GVHD of mouth and skin was associated with the occurrence of ocular GVHD at 3 months after transplantation [16]. However, their finding of matched related donor allogeneic HSCT as a risk factor was not observed in the present study (Table 4). Since small sample size limits multivariate analysis, further studies with larger sample sizes may clarify this issue.

The mainstay of therapy for conjunctival aGVHD includes lubrication, topical antibiotics and topical anti-inflammatory agents [27]. Topical corticosteroids promote lymphocyte apoptosis and suppress cell-mediated inflammation [28–29]. However, some studies have reported no effect of topically applied corticosteroids for pseudomembranous conjunctivitis [17–18]. Due to the small sample size of pseudomembranous conjunctivitis, it is hard to analyze the effect of topical corticosteroids in our study. Our clinical experience is in agreement with previous studies [17–18]. We found that topical corticosteroids do not halter pseudomembranous formation. In contrast, the course of conjunctivitis is correlated with the course of systemic aGVHD in response to treatment. Once systemic aGVHD is dampened by the increasing intensity of systemic immunosuppressive therapy, pseudomembrane formation starts to ameliorate.

In the past, a- and cGVHD were arbitrarily defined by the events occurring either before or 100 days post transplantation. Current NIH consensus criteria for definitions are based on phenotype rather than on the timing of GVHD occurrence after HSCT or DLI [20]. A diagnostic criterion for ocular cGVHD was also defined [20]. However, there is no standardized diagnostic criterion for ocular aGVHD. In the current study, the definition of conjunctival aGVHD was not based on timing. We think our definition would not engender debate since it correlates with NIH categories for aGVHD. Nevertheless, a diagnostic criterion for ocular aGVHD is warranted for clinical trials in the future.

Owing to the low incidence of conjunctival aGVHD, our study was limited by the small sample size. Because this is a retrospective review, it is possible that some patients with ocular findings were missed because of lack of severity of reported symptoms. However, the incidence of conjunctival aGVHD in our study is correlated to those in other studies, so the possibility of missing cases might be low. Nevertheless, a prospective study with regular ophthalmological evaluation is required to validate the correlation between conjunctival aGVHD and systemic prognosis.

Studies for ocular aGVHD are scarce because the incidence is lower than that of ocular cGVHD. Besides, to avoid unnecessary examination in ill patients immediately after transplantation, an ophthalmologist is usually not routinely consulted at the acute stage. Of note, conjunctival aGVHD may lead to corneal complications, and conjunctival scarring, resulting in ocular surface disturbance and dry eye in the future, which largely impair quality of life and activities of daily living [9, 12, 16, 24]. To avoid these morbidity, an ophthalmologist should be consulted at the acute stage if patients have any ocular symptoms or signs. Although conjunctival aGVHD is not correlated with survival, the close correlation with systemic GVHD reminds oncologists of the need to adjust the dosage of immunosuppressive agents.

## Supporting Information

S1 Table Risk factors for conjunctival acute graft-versus-host disease after adjusted competing mortality. (DOCX)

S1 Data Minimal data. Data of patients with conjunctival acute graft-versus-host disease.
(XLS)
