# Supplementary material for: Conjunctival Acute Graft-versus-Host Disease in Adult Patients Receiving Allogeneic Hematopoietic Stem Cell Transplantation: A Cohort Study
Source: PLoS One. 2016 Nov 30;11(11):e0167129. doi: 10.1371/journal.pone.0167129 (PMC5130243; doi:10.1371/journal.pone.0167129)
Supplement: S1 Table — (DOCX) [file pone.0167129.s001.docx]

| **Supplemental table 1. Risk factors for conjunctival aGVHD after adult allogeneic HSCT after adjusted competing mortality** | | | | | | | | | | |
| --- | --- | --- | --- | --- | --- | --- | --- | --- | --- | --- |
| **Factors** | ***No. of***  ***patients*** | ***Conjunctival aGVHD*** | | **Univariate analysis** | | | **Multivariate analysis** | | |  |
|  |  | *n* | **Per 10,000 person-day** | *SHR* | *95% CI* | *P* value | *SHR* | *95% CI* | *P* value |  |
| **Age at HSCT** |  |  |  |  |  |  |  |  |  |  |
| ≤ 55 | 105 | 8 | 1.6 |  |  |  |  |  |  |  |
| > 55 | 34 | 5 | 4.2 | 2.020 | 6.151–2.020 | 0.216 |  |  |  |  |
| **EBMT risk score** |  |  |  |  |  |  |  |  |  |  |
| ≤ 4 | 105 | 8 | 1.6 |  |  |  |  |  |  |  |
| > 4 | 34 | 5 | 4.4 | 1.877 | 5.790–1.877 | 0.273 |  |  |  |  |
| **Sex** |  |  |  |  |  |  |  |  |  |  |
| Female | 66 | 9 | 3.0 |  |  |  |  |  |  |  |
| Male | 73 | 4 | 1.3 | 0.414 | 1.321–0.414 | 0.136 |  |  |  |  |
| **Disease type** |  |  |  |  |  |  |  |  |  |  |
| Myeloid | 78 | 8 | 2.6 |  |  |  |  |  |  |  |
| Non-myeloid | 61 | 5 | 1.6 | 0.646 | 1.889–0.646 | 0.425 |  |  |  |  |
| **Conditioning** |  |  |  |  |  |  |  |  |  |  |
| Reduced-intensity | 55 | 5 | 2.1 |  |  |  |  |  |  |  |
| Myeloablative | 84 | 8 | 2.1 | 1.106 | 3.419–1.106 | 0.862 |  |  |  |  |
| **Conditioning** |  |  |  |  |  |  |  |  |  |  |
| Other | 90 | 4 | 1.5 |  |  |  |  |  |  |  |
| TBI-based | 49 | 9 | 2.5 | 1.370 | 4.313–1.370 | 0.590 |  |  |  |  |
| **Conditioning** |  |  |  |  |  |  |  |  |  |  |
| Other | 101 | 9 | 1.9 |  |  |  |  |  |  |  |
| Fludarabine-based | 38 | 4 | 2.7 | 1.084 | 3.632–1.084 | 0.897 |  |  |  |  |
| **Transplant no.** |  |  |  |  |  |  |  |  |  |  |
| One | 122 | 12 | 2.2 |  |  |  |  |  |  |  |
| Multiple | 17 | 1 | 1.2 | 0.427 | 2.571–0.427 | 0.353 |  |  |  |  |
| **Donor type** |  |  |  |  |  |  |  |  |  |  |
| Matched sibling | 54 | 5 | 1.8 |  |  |  |  |  |  |  |
| Alternative donors | 85 | 8 | 2.4 | 1.177 | 3.531–1.177 | 0.771 |  |  |  |  |
| **aGVHD grade*** |  |  |  |  |  |  |  |  |  |  |
| 0-II | 106 | 5 | 1.0 |  |  |  |  |  |  |  |
| III-IV | 33 | 8 | 6.7 | 4.639 | 13.761–4.639 | 0.006 | 1.431 | 9.638–1.431 | 0.713 |  |
| **aGVHD of skin** |  |  |  |  |  |  |  |  |  |  |
| Stage 0-I | 101 | 3 | 0.6 |  |  |  |  |  |  |  |
| Stage II-IV | 38 | 10 | 6.6 | 8.108 | 28.677–8.108 | 0.001 | 6.449 | 57.944–6.449 | 0.096 |  |
| **CMV infection** |  |  |  |  |  |  |  |  |  |  |
| No | 45 | 3 | 1.5 |  |  |  |  |  |  |  |
| Yes | 94 | 10 | 2.4 | 1.548 | 5.351–1.548 | 0.489 |  |  |  |  |
| **Abbreviations:** CI = confidence interval; SHR = subdistribution hazard ratio; EBMT = European Group for Blood and Marrow Transplantation; HSCT = hematopoietic stem cell transplantation; GVHD = graft-versus-host disease; aGVHD = acute GVHD; cGVHD = chronic GVHD; CMV = cytomegalovirus; TBI = total body irradiation; No. = number (s). Factors with statistical significance (*p* < 0.1) upon univariate analysis were included in multivariate analysis  *:overall grade of aGVHD except skin involvement | | | | | | | | | | |
